# High-dose Vitamin-B6 reduces sensory over-responsivity

**DOI:** 10.1177/02698811241271972

**Published:** 2024-08-24

**Authors:** Rebekah O Cracknell, Teresa Tavassoli, David T Field

**Affiliations:** School of Psychology and Clinical Language Sciences, The University of Reading, Reading, Berkshire, UK

**Keywords:** GABA, glutamate, excitation–inhibition balance, autism, sensory hyperreactivity

## Abstract

**Background:**

Sensory reactivity differences are experienced by between 5% and 15% of the population, often taking the form of sensory over-responsivity (SOR), in which sensory stimuli are experienced as unusually intense and everyday function is affected. A potential mechanism underlying over-responsivity is an imbalance between neural excitation and inhibition in which inhibitory influences are relatively weakened. Therefore, interventions that boost neural inhibition or reduce neural excitation may reduce SOR; Vitamin-B6 is the coenzyme for the conversion of excitatory glutamate to inhibitory gamma-aminobutyric acid (GABA), and in animal models, it both increases the concentration of GABA and reduces glutamate.

**Aims:**

To discover whether taking a high dose of Vitamin-B6 reduces SOR and other aspects of sensory reactivity.

**Methods:**

We recruited 300 adults (249 females) from the general population who completed the Sensory Processing 3-Dimensions Scale (SP-3D) first at baseline, and again following randomisation to either 1 month’s supplementation with 100 mg Vitamin-B6, or one of two control conditions (1000 µg Vitamin-B12 or placebo). To focus on individuals who experience SOR, we analysed the effects of supplementation only on individuals with high baseline SOR scores (above the 87th percentile).

**Results:**

In individuals with SOR at baseline, Vitamin-B6 selectively reduced SOR compared to both placebo and Vitamin-B12. We also found that Vitamin-B6 selectively reduced postural disorder in individuals with high scores on this subscale at baseline, but there were no effects on the four remaining SP-3D subscales.

**Conclusions:**

Clinical trials and mechanistic studies should now be conducted in autism, attention deficit hyperactivity disorder and other groups with SOR.

## Introduction

Sensory reactivity differences, such as sensory over-responsivity (SOR) aka hyperreactive (e.g. being overwhelmed by everyday sounds), sensory under-responsivity (SUR) aka hyporesponsive (e.g. showing no response when touched) or sensory seeking (SR) (e.g. being fascinated by moving objects), are seen in ~65%–90% of autistic individuals, ~30% of children with special educational needs and ~10%–15% of children overall (Ben-Sasson et al., 2019; Tavassoli et al., 2016). Attention deficit hyperactivity disorder (ADHD) presents a similar picture, with one study estimating 43% co-occurrence in females with ADHD and 22% in males with ADHD (Bijlenga et al., 2017). The prevalence of sensory reactivity differences in the absence of co-occurring conditions has been estimated at 5% (Galiana-Simal et al., 2020). They impact developmental outcomes and quality of life (Dunn, 1997) as well as mental well-being (MacLennan et al., 2020, 2021; Rossow et al. 2021a, 2021b).

Of the three facets of sensory reactivity differences, work on underlying causal mechanisms is most advanced in the case of sensory hyperreactivity, which we will focus on here, and the evidence points to a potential difference in the balance between glutamatergic excitation and GABAergic inhibition (E-I balance). For example, investigations of tactile SOR using magnetic resonance spectroscopy have revealed reduced GABA concentrations in the sensorimotor cortex of both autistic children and adults (Gaetz et al., 2014; Puts et al., 2016; Sapey-Triomphe et al., 2019). More recently, Wood et al. (2021) found reduced thalamic GABA concentrations in autistic youth that were related to behavioural SOR symptoms, as well as atypical thalamic connectivity with sensory processing regions. However, not all studies have found GABAergic differences in autism (Kolodny et al., 2020), and one recent study instead found elevated glutamate in primary sensorimotor cortex, which correlated positively with parent-reported SOR (He et al., 2021). Although superficially paradoxical, the latter findings are consistent with the E-I balance theory of sensory symptoms in autism because the consequences of increased excitation may be similar to those of reduced inhibition (Rubenstein and Merzenich, 2003). In the case of SUR, the cause may be dysregulation of glutamatergic neurotransmission: Phelan-McDermid Syndrome produces disruption in the *SHANK3* gene, which encodes a scaffolding protein in glutamatergic synapses, and produces a specific autistic phenotype in which SUR is considerably more prevalent than in idiopathic autism (Tavassoli et al., 2021). While the picture is complex and details are still emerging, it is clear that sensory reactivity differences are related to the E-I balance (Foss-Feig et al., 2017; Rosenberg et al., 2015).

The relationship between E-I balance and the presence of SOR suggests that interventions that boost GABAergic tone and/or reduce glutamatergic excitation may reduce the intensity of SOR. Consistent with this, some pharmacological interventions that do this have produced encouraging results in clinical trials performed with autistic participants, for example, Bumetanide (Canitano and Palumbi, 2021). Here, we propose that Vitamin-B6 taken at pharmacological doses is an intervention with the potential to alter E-I balance. This is plausible because it is well established that the biologically active form of Vitamin-B6, pyridoxal-5′-phosphate (PLP), acts as a coenzyme for glutamate decarboxylase (GAD), which catalyses the conversion of glutamate to gamma-aminobutyric acid (GABA) in the brain (e.g. Martin and Rimvall, 1993; Petroff, 2002). If increasing the availability of the coenzyme, PLP, were to increase the rate of this reaction, the result would be a shift in the balance of concentrations between the two neurotransmitters, with a consequent relative increase in the influence of inhibition on the information processing performed by neural networks. Because most of the enzyme GAD exists in the inactive apoenzyme form that is not bound to the coenzyme, PLP, the reaction that converts glutamate to GABA is not saturated under normal physiological conditions, and there is therefore considerable potential to increase its rate by boosting PLP (Martin and Rimvall, 1993; Petroff, 2002; Sharma et al., 1994). Providing proof of concept for this, Sharma et al. (1994) compared GABA and glutamate concentrations in the brains of Vitamin-B6-deficient rats to rats fed a high-Vitamin-B6 diet and found that Vitamin-B6 deficiency substantially reduced GABA concentration and increased glutamate concentration compared to the high-B6 diet. A recent study performed in mice (Walia et al., 2018) confirmed that supplementary B6, as opposed to deficiency, can drive this effect; three acute dose levels were tested, using intraperitoneal injection, and only the intermediate dose reduced anxiety and glutamate concentration while increasing GABA. This suggests that careful work will be required to establish the range of doses that are effective in humans.

Given that the concentration of the activated form of Vitamin-B6, PLP, is an important determinant of the rate of production of GABA, it is interesting to note that there are substantial differences between individuals in the ability to metabolise inactive dietary Vitamin-B6 into the active form. This may be caused by genetically determined variation in the binding affinity of enzymes such as pyridoxal kinase and pyridoxine-5′-phosphate oxidase that participate in the Vitamin-B6 salvage pathway (Ames et al., 2002). Furthermore, studies of Vitamin-B6 status in autism have found either reduced PLP levels or dramatic variability in PLP levels between autistic individuals relative to non-autistic individuals (Audhya, 2004; Adams et al., 2011); we speculate that this is connected to the high prevalence of SOR in autism. Furthermore, significant individual differences in Vitamin-B6 metabolism have also been demonstrated in the general population by pharmacokinetic studies (Hadtstein & Vrolijk., 2021; Vrolijk et al., 2020). It is likely that those individuals whose ability to convert dietary Vitamin-B6 into active PLP is lower are at greater risk of not being able to regulate GABA production effectively, and it is these individuals that are most likely to benefit from the high-dose Vitamin-B6 intervention tested here (Ames et al., 2002).

Taken together, the evidence we have reviewed suggests that high-dose Vitamin-B6 supplementation should produce changes at the behavioural level consistent with increased neural inhibition. Testing this prediction, Field et al. (2022) supplemented an opportunity sample of students and members of the public with 100 mg of Vitamin-B6, corresponding to between 50 and 100 times the typical dietary intake, as pyridoxine, for 1 month. A range of behavioural outcomes broadly linked to neural inhibition was measured, and the key findings supporting the prediction were that the strength of visual surround suppression—an index of GABAergic lateral inhibition in the visual cortex—was increased by Vitamin-B6, while anxiety was reduced.

To test the current prediction that SOR will be reduced by high-dose Vitamin-B6 supplementation, we recruited a general population sample of 300 participants and randomised them to receive 100 mg of Vitamin-B6 as pyridoxine, placebo or 1000 mg of Vitamin-B12, daily for 1 month. Vitamin-B12 was included because data were collected as part of a larger project with multiple outcome measures. Here, it acts as an additional control condition. To quantify SOR at baseline, and again at follow-up, participants completed the Sensory Processing 3-Dimensions Scale (SP-3D), which includes an SOR subscale. To focus our analysis on individuals who experience SOR, we analysed the effects of supplementation only on individuals with high baseline SOR scores falling at or above the 87th percentile. The SP-3D also includes subscales for several traits that often co-occur with SOR: SUR, SR, discrimination (SD) and motor control issues related to sensory processing—dyspraxia and postural disorder (PD). To test whether the effect of Vitamin-B6 was specific to SOR, we repeated our analysis procedure for these subscales, in each case selecting participants with high scores on the subscale at baseline.

## Methods

### Participants

The larger project, of which the data reported here are a subset, was conducted in six phases by successive cohorts of BSc and MSc students (phases 1–5) and a doctoral student (phase 6). Participants were recruited in exchange for course credit via adverts, word of mouth and social media. Participants in phase 6 only were paid £30 to complete the study. In this way, a total of 649 were randomised to receive either placebo tablets (198 participants), Vitamin-B6 (229 participants), or Vitamin-B12 (222 participants). To provide some flexibility in the scheduling of follow-up laboratory visits, the period of supplementation was allowed to vary between 30 and 40 days. While the experimental manipulation remained constant throughout the project, no single outcome measure was included in all phases of data collection: the SP-3D questionnaire data reported here were collected in phases 4–6, from a total of 300 participants (249 females, 51 males; aged 18–60 years, mean 25.8, median 21.8, SD 9.6). Of these, 81 were randomised to receive a placebo, 113 to Vitamin-B6 and 106 to Vitamin-B12. For more detailed information about the tablets used, the double-blinding methods and the full set of outcomes measured across the phases of the project, see Field et al. (2022), who report on this in detail for phases 1–5 (phase 6 procedures were identical to phase 5 procedures: 171 participants took part in phase 6). Note that phases 1–4 were conducted in person, while phases 5–6 were carried out online, which required posting supplements to participants. Data collected from participants who took part in phases 4 and 5 are reported both here and in Field et al. (2022), but the outcome measures reported are different: SP-3D questionnaire scores are only reported here.

To take part, participants had to confirm that they were not lactose intolerant, diabetic, epileptic or taking medications known to interfere with Vitamin-B absorption. They were also asked if they were taking supplements that contained Vitamin-B and were excluded unless they agreed to stop taking them for the duration of their participation in the study. The study was approved by the University of Reading Ethics Committee (2018-128-DF). Written informed consent was obtained from all participants, and an online tick box replaced the requirement for a signature in phases 5 and 6.

### Sensory processing scale questionnaire

The Sensory Processing Scale Inventory self-inventory, aka SenSOR and SP-3D (Miller et al., 2017; Schoen et al., 2016; Mulligan et al., 2019; Verhulst et al., 2022) consists of six subscales with varying numbers of questions per scale: SOR (12 items, example: *Some types of visual sensory input bother me, such as fluorescent lights, sunlight, visually cluttered environments*), SUR (7 items, example: *I do not respond to touch experiences, such as leave clothing twisted on body, do not notice dirty or wet clothes or messy hands*), sensory craving (SC, 10 items, example: *I cannot stop watching visual stimuli, especially contrasting or moving objects, such as spinning objects like wheels, fast-changing images on screens, rotating fans*), PD (7 items, example: *I have poor posture and often slump at a desk or table or lean against walls or furniture*), dyspraxia (13 items, example: *I have trouble with daily tasks involving use of hands/fingers, such as handling utensils, dressing, managing fasteners on clothes, eating neatly without making a mess on the table/floor or self*), and sensory discrimination (SD, 10 items, example: *I have difficulty understanding what is said or distinguishing between similar sounds or words, such as cat vs. cap, back vs. bat*). Each question is answered yes or no. Because the maximum score on each subscale varies, we divided each score by the maximum possible score on the respective subscale. This resulted in all subscales having a minimum score of 0 and a maximum of 1.

### Data analysis

To focus our analysis on individuals who experience SOR, we had intended to analyse the effects of supplementation only on individuals in our sample whose baseline SOR scores fell above the 90th percentile of the scores in the full sample. In practice, because there are only 12 possible scores on the SOR subscale we divided the sample at the 87th percentile. As an exploratory analysis, and to discover whether the effect of Vitamin-B6 was specific to SOR, we repeated the analysis procedure for the other subscales of the SP-3D, in each case cutting the sample just below the 90th percentile.

To test the prediction that Vitamin-B6 supplementation would be beneficial for SOR, as well as for the exploratory analyses, we used a 2 (baseline vs post-test) by 3 (treatment group) mixed analysis of variance (ANOVA), followed up by paired samples *t*-tests assessing change between baseline and post-test within each treatment group. Whenever ANOVA revealed a significant interaction, if the interpretation of the interaction was not fully clarified by the *t*-tests, we further followed up with 2 * 2 ANOVAs as needed. In any design that selects participants on the basis that they score highly on a particular trait or state at baseline, there is likely to be some regression towards the central tendency of the measurement scale over time (Barnett, 2005). Therefore, we expected to see some reduction in SOR scores at follow-up in all three experimental groups, and this effect could potentially be additive with placebo effects. Given this, only patterns of results in which one experimental group yielded a significantly greater reduction in scores than the other two, or where one of the three groups produced a significant change in scores but the other two did not would allow us to conclude that a treatment effect had occurred.

Because we extracted six different subsets of data from the full sample, this raises the statistical question of the degree of independence of the six subsamples. We therefore quantified the extent to which the six subsamples overlapped with each other. The variation in the degree of overlap between pairs of subsamples was also of interest for comparison with previous studies that have quantified the co-occurrence of different aspects of sensory reactivity differences. To do this, for each of the 15 possible pairings (e.g. SOR with SUR, SOR with SC, SOR with SC), we calculated the percentage of participants selected for both subsets as a percentage of the total number of participants in either subset. For example, the total number of people with both SOR and SUR was divided by the sum of the number of people reporting SOR and the number of people reporting SUR.

## Results

### Vitamin-B6 reduces sensory hyperreactivity

**Figure 1.**
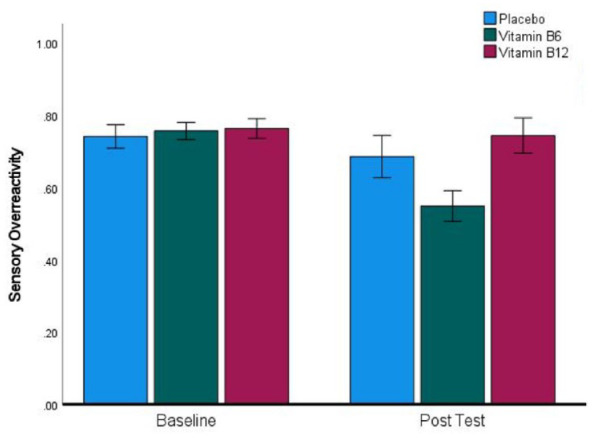
A significant reduction in sensory over-reactivity scores in the Vitamin-B6 group between baseline and post-test. Error bars indicate ±1 SEM.

Our key prediction was that sensory hyperreactivity would be reduced by high-dose Vitamin-B6. 13% of participants scored above the 87th percentile at baseline, which corresponded to a score of 0.67 or higher on the standardised SOR subscale, where the maximum possible score was 1, and the minimum possible score was 0. Participants in the high SOR subgroup endorsed a minimum of 8 out of 12 yes/no items on the SOR subscale of the SP-3D, and the mean number of items endorsed in this group was 9.36 out of 12. This indicates that we were successful in finding participants who experience SOR, although we were not able to find published norms for this version of the SP-3D to compare with the scores in our sample. Within this group of 39 individuals, 9 had been randomised to placebo, 17 to Vitamin-B6 and 13 to Vitamin-B12. Figure 1 presents the SOR scores of the three treatment groups at baseline and post-test, revealing that as predicted SOR scores fall more at post-test in the Vitamin-B6 group than in the other groups. This pattern was confirmed by a highly significant interaction (*F*(2, 36) = 5.92, *p* = 0.006, η_p_^2^ = 0.247). Following this up with *t*-tests revealed a non-significant reduction in the Vitamin-B12 group (*t*(12) = 0.64, *p* = 0.53, *d* = 0.18), a marginally significant reduction in the placebo group (*t*(8) = 2.3, *p* = 0.05, *d* = 0.74), and a highly significant reduction in the Vitamin-B6 group (*t*(16) = 4.04, *p* < 0.001, *d* = 0.98). To test directly whether the reduction in SOR was reliably greater for Vitamin-B6 than placebo, we carried out a 2 * 2 ANOVA, which resulted in a significant interaction confirming that this was the case (*F*(1, 24) = 4.29, *p* = 0.049, η_p_^2^ = 0.152).

#### Effects of cohort characteristics

**Figure 2.**
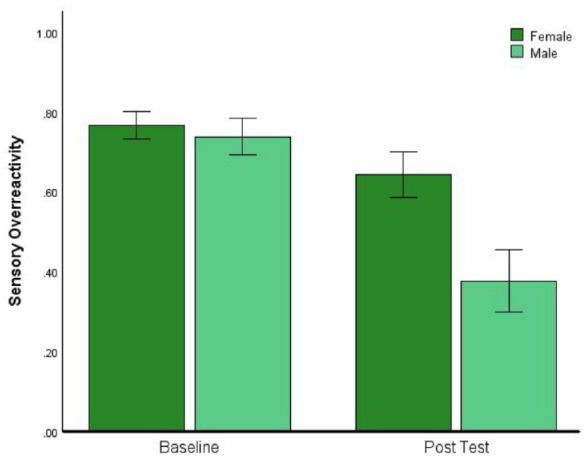
Male participants who took Vitamin-B6 experienced a greater reduction in sensory reactivity than females. Error bars indicate ±1 SEM.

The proportion of participants in the high SOR subgroup who were male (20.5%) was slightly higher than the proportion who were male in the remaining 87% of the total sample (16.5%); however, this did not approach difference when tested by Chi-square, (*χ*^2^ (1) = 0.39, *p* = 0.531). We also conducted ANOVA to ask whether the reduction of SOR by Vitamin-B6 in the high SOR subgroup was modulated by sex. Selecting only the 11 female and 6 male participants who were both in the high SOR subgroup and had been randomised to receive Vitamin-B6, we performed a 2 (baseline vs post-test) by 2 (female vs male) ANOVA: see Figure 2 for a plot of means with error bars. This reveals a larger reduction in SOR in male participants, confirmed by a significant interaction (*F*(1, 15) = 6.60, *p* = .021, η_p_^2^ = 0.306). Following this up with *t*-tests revealed that the reduction in SOR was statistically significant in both the female and male subgroups (*t*(10) = 3.08, *p* = 0.012, *d* = 0.93; *t*(10) = 3.49, *p* = 0.018, *d* = 1.42).

We also compared the age distribution of the high SOR subgroup with the age distribution of the total sample and found that these were similar: full sample mean 25.8, median 21.8, SD = 9.6; high SOR mean 27.4, median, 22, SD = 11.7. Confirming that age was not related to SOR scores in our sample, Spearman’s correlation between the two was close to zero (*r*(298) = 0.033, *p* = 0.57).

### Vitamin-B6 reduces PD

**Figure 3.**
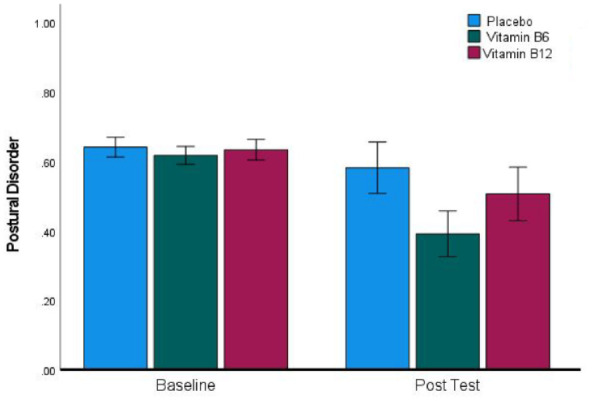
A significant reduction in postural disorder scores in the Vitamin-B6 group between baseline and post-test. Error bars indicate ±1 SEM.

To find out whether the effect of Vitamin-B6 was selective to SOR or broader in nature, we analysed the other subscales of the SP-3D; a summary of the resulting means, standard deviations and sample sizes is presented in Table 1. Only in the case of PD was there a reduction in scores in the Vitamin-B6 group that was unlikely to be explainable by placebo effects or regression to the mean. 12.6% of participants scored 0.57 or higher at baseline, implying that they had endorsed 4 or more items out of 7 on the PD subscale, and the mean number of items endorsed in this group was 4.4 out of 7. Within this group of 38 individuals, 12 had been randomised to placebo, 15 to Vitamin-B6 and 11 to Vitamin-B12. The PD scores of the three treatment groups at baseline and post-test are presented in Figure 3, which reveals a general tendency towards reduction in PD scores at post-test that is larger for Vitamin-B6. While the larger effect in the Vitamin-B6 group did not result in a significant interaction (*F*(2, 35) = 1.47, *p* = 0.243, η_p_^2^ = 0.078), the main effect of reduction at post-test was highly significant (*F*(1, 35) = 10.9, *p* = 0.002, η_p_^2^ = 0.238). Investigating this further, only for Vitamin-B6 did follow-up *t*-tests reveal a significant reduction (*t*(14) = 3.20, *p* = 0.006, *d* = 0.83), while the change from baseline was non-significant in the placebo (*t*(11) = 1.11, *p* = 0.293, *d* = 0.32) and Vitamin-B12 groups (*t*(10) = 1.46, *p* = 0.174, *d* = 0.44).

**Table 1.** Details of the sub-samples isolated from the main sample, for each of the facets of sensory integration difference in the SP-3D. To facilitate comparison between subscales, scores were transformed to vary between 0 and 1.

Subscale	*N*			Baseline means (SD)			Post-test means (SD)		
	Placebo	B6	B12	Placebo	B6	B12	Placebo	B6	B12
SOR	9	17	13	0.74 (0.06)	0.76 (0.11)	0.76 (0.10)	0.68 (0.08)	0.55 (0.23)	0.74 (0.14)
SUR	7	12	12	0.45 (0.05)	0.56 (0.14)	0.50 (0.17)	0.33 (0.13)	0.33 (0.26)	0.36 (0.17)
SC	7	15	12	0.41 (0.17)	0.47 (0.24)	0.39 (0.33)	0.43 (0.11)	0.37 (0.26)	0.33 (0.27)
SD	10	16	16	0.34 (0.07)	0.44 (0.16)	0.40 (0.16)	0.25 (0.06)	0.26 (0.24)	0.30 (0.16)
PD	12	15	11	0.64 (0.13)	0.62 (0.09)	0.63 (0.07)	0.58 (0.22)	0.63 (0.07)	0.51 (0.31)
Dyspraxia	15	18	11	0.43 (0.09)	0.56 (0.19)	0.47 (0.12)	0.31 (0.18)	0.39 (0.21)	0.47 (0.12)

### No effects of Vitamin-B6 on other aspects of sensory reactivity

Turning to SUR, a general reduction in scores occurred at the post-test, but this was not selective to Vitamin-B6. Participants were included in the analysis if their baseline SUR score was 0.43 or higher. Only the main effect of baseline versus post-test in the ANOVA reached significance (*F*(1, 28) = 25.18, *p* < 0.001, η_p_^2^ = 0.473), and this was driven by significant reductions at post-test in all three treatment conditions: placebo (*t*(6) = 2.51, *p* = 0.046, *d* = 0.95), Vitamin-B6 (*t*(11) = 3.35, *p* = 0.006, *d* = 0.97) and Vitamin-B12 (*t*(11) = 4.03, *p* = 0.002, *d* = 1.16). This pattern is clearly consistent with regression to the mean.

In the case of SD, scores reduced at post-test, but placebo and regression to the mean effects cannot be ruled out as an explanation of this. 13% of participants scored 0.21 or higher on this scale at baseline and were included in the analysis. Only the main effect of baseline versus post-test in the ANOVA reached significance (*F*(1, 39) = 11.69, *p* = 0.002, η_p_^2^ = 0.230), and this was driven not only by a Vitamin-B6 effect (*t*(15) = 2.65, *p* = 0.018, *d* = 0.66) but also by an effect approaching significance for Vitamin-B12 (*t*(15) = 1.96, *p* = 0.069, *d* = 0.49), and a non-significant reduction in the placebo group (*t*(9) = 1.55, *p* = 0.156, *d* = 0.49).

For the SC subscale, participants were included in the analysis if their SC score was .4 or higher (11.3% of the sample). Scores did not change in the placebo group and were lower at post-test in both the Vitamin-B6 and B12 groups, but with only seven participants in the placebo group, we cannot rule out that the changes in the other two groups were driven by a mixture of regression to the mean and placebo effects. Statistically, this was reflected in a highly significant main effect of baseline versus post-test in the ANOVA, (*F*(1, 31) = 10.45, *p* = 0.003, η_p_^2^ = 0.252), a non-significant *t*-test in the placebo condition (*t*(6) = 0.65, *p* = 0.54, *d* = 0.25), and significant *t*-tests in both the Vitamin-B6 condition (*t*(14) = 2.49, *p* = 0.026, *d* = 0.64) and the Vitamin-B12 condition (*t*(11) = 3.11, *p* = 0.01, *d* = 0.90).

Participants were included in the Dyspraxia subscale analysis if their score was 0.31 (14% of the sample). Reduced scores at post-test were evident in both the placebo and Vitamin-B6 groups, which does not permit us to rule out placebo and regression to the mean as explanations of these effects. Statistically, this produced a highly significant main effect of baseline versus post-test in the ANOVA, (*F*(1, 41) = 15.30, *p* < 0.001, η_p_^2^ = 0.272), significant *t*-tests in both the placebo (*t*(14) = 2.58, *p* = 0.022, *d* = 0.67) and Vitamin-B6 (*t*(17) = 3.63, *p* = 0.004, *d* = 0.79) conditions. and a non-significant difference in the B12 condition (*t*(10) = 1.14, *p* = 0.282, *d* = 0.34).

### Effects of Vitamin-B6 on sensory reactivity in the remainder of the sample

To provide a more comprehensive view of the data, and to check whether the effects of high-dose Vitamin-B6 we observed in the high SOR and high PD subgroups were also present in the rest of the sample whose baseline scores were lower, we repeated all of the *t*-tests reported in sections ‘Vitamin-B6 reduces sensory hyperreactivity’, ‘Vitamin-B6 reduces postural disorder’ and ‘No effects of Vitamin-B6 on other aspects of sensory reactivity’ above using reversed inclusion criteria. The means, standard deviations and sample sizes submitted to this analysis are presented in Table 2; inspection of these descriptives reveals a trend towards reduction of SOR at post-test, but this was present in all three groups including placebo. It also reveals a selective trend towards a reduction in PD scores in the B6 group. However, none of these trends reached statistical significance in the *t*-tests we performed, despite the greater power to detect small effect sizes afforded by the larger sample sizes used for these analyses.

**Table 2.** Descriptive statistics for the remainder of the main sample after the exclusion of high-scoring individuals, for each of the facets of sensory integration difference in the SP-3D. To facilitate comparison between subscales comprised of different numbers of questions, scores were transformed to vary between 0 and 1.

Subscale	*N*			Baseline means (SD)			Post-test means (SD)		
	Placebo	B6	B12	Placebo	B6	B12	Placebo	B6	B12
SOR	72	96	93	0.30 (0.15)	0.27 (0.19)	0.25 (0.16)	0.27 (0.18)	0.25 (0.21)	0.22 (0.17)
SUR	74	101	94	0.11(0.12)	0.09 (0.11)	0.09 (0.11)	0.11 (0.14)	0.09 (0.14)	0.08 (0.12)
SC	69	94	97	0.10 (0.10)	0.08 (0.10)	0.01 (0.10)	0.12 (0.16)	0.08 (0.11)	0.11 (0.14)
SD	71	97	90	0.06 (0.07)	0.05 (0.06)	0.05 (0.07)	0.09 (0.13)	0.06 (0.11)	0.06 (0.11)
PD	69	98	95	0.17 (0.16)	0.17 (0.16)	0.15 (0.14)	0.17 (0.20)	0.15 (0.18)	0.15 (0.16)
Dyspraxia	66	95	95	0.15 (0.09)	0.14 (0.10)	0.14 (0.10)	0.15 (0.13)	0.15 (0.14)	0.15 (0.16)

### Co-occurrence of the six aspects of sensory integration differences

The six subsets of the full sample reported above were partially dependent on each other, and the observed degree of dependence varied substantially between the subscales of the SP-3D. Table 3 breaks down the dependence by subscale pairs, and, in general, this is much greater than would be expected by chance (approximately 10%). The mean overlap between the six selected subsets of the full sample was 23.78%, and 107 of the 300 participants tested met the threshold for inclusion in at least one of the subsets analysed above. Relatively much higher overlaps were observed between SUR and SC, and between PD and dyspraxia, while relatively much lower overlaps were observed between SOR and SUR, as well as between SOR and SC.

**Table 3.** Co-occurrence of the six facets of sensory integration difference measured by the SP-3D questionnaire, indicated by the percent overlap between subgroups reporting high levels of each facet.

Subscale	SOR	SUR	SC	SD	PD
SOR					
SUR	14.8				
SC	14.1	58.6			
SD	22.7	19.7	20.6		
PD	22.2	16.9	18.0	27.0	
Dyspraxia	25.8	17.2	16.4	28.3	34.4

## Discussion

We described a range of evidence suggesting that high-dose Vitamin-B6 changes the excitation–inhibition balance in the brain. The strongest evidence comes from rat and mouse models, in which manipulating Vitamin-B6 availability in the brain increases inhibitory GABA concentration, as well as reducing the concentration of the excitatory neurotransmitter, glutamate (Sharm et al., 1994; Walia et al., 2018). Because sensory hyperreactivity has been linked to reduced GABAergic tone and altered E-I balance, we predicted that it would be improved by taking a high dose of Vitamin-B6. We confirmed this prediction in a group of individuals with sensory hyperreactivity, who were selected from a larger opportunity sample of the general population. The questionnaire we used to measure sensory hyperreactivity, the SP-3D, contained five additional subscales measuring other sensory problems and related motor control and postural problems that can co-occur with sensory hyperreactivity. These subscales, along with the inclusion of an extra group of participants who took a high dose of Vitamin-B12, allowed us to ask whether the effect of Vitamin-B6 on sensory hyperreactivity is selective. We found no effects of Vitamin-B12 compared to placebo, while Vitamin-B6 also improved scores on the PD subscale, suggesting that high-dose Vitamin-B6 may also be of benefit for motor control problems that commonly co-occur with sensory hyperreactivity.

Altered E-I balance plays a role in a wide range of conditions, including epilepsy (Staley, 2015), anxiety and depression (Luscher and Fuchs, 2015; Sohal and Rubenstein, 2019), schizophrenia (Lang et al., 2007), autism (Rubenstein and Merzenich, 2003), migraine (Vecchia and Pietrobon, 2012), ADHD (Mamiya et al., 2021), and obsessive-compulsive disorder (Biria et al., 2023). One common factor across these conditions is SOR (Bijlenga et al., 2017; Budnick & Braff, 1992; Engel-Yeger et al., 2016; Lewin et al., 2015; Pearl et al., 2020; Van Campen et al., 2015). Our results, along with those of Field et al. (2022), suggest that high-dose Vitamin-B6 should be tested as an intervention for sensory over-responsivity in each of these conditions. Because E-I balance differences may also underly other features of these conditions in addition to SOR, the benefits of high-dose B6 may extend beyond a reduction in SOR. However, future studies must take great care to establish the effective dose range because mouse models have revealed an n-shaped function in which lower and higher doses of Vitamin-B6 did not influence GABA or glutamate concentrations (Walia et al., 2018). The n-shaped dose–response curve could account for the mixed results of previous studies that tested the effect of a single dose level of Vitamin-B6, which in some cases was either a ‘mega dose’ 10 or 20 times greater than used here, or a smaller dose than the 100 mg used here, to produce beneficial effects in autism (Li et al., 2018; Murza et al., 2010). Furthermore, it is well established that very high doses of Vitamin-B6 taken as pyridoxine, when sustained over time, are toxic, causing peripheral neuropathy (Schaumburg et al., 1983). More recently, it has become apparent that in a small minority of individuals differences in the metabolic processes converting pyridoxine to the active form (PLP) can result in peripheral neuropathy occurring at much lower doses (Vrolijk et al., 2017). Therefore, high-dose Vitamin-B6 is an intervention that should be supervised by medical professionals. There are promising indications that dietary supplementation with Vitamin-B6 as pyridoxal (PL) or PLP eliminates the risk of peripheral neuropathy (Vrolijk et al., 2020), but caution is required until large-scale clinical trials have been conducted.

### Relationship to existing neurobiological models of SOR

Functional brain imaging studies have revealed that autistic individuals with SOR have greater neural responses to sensory stimulation in primary sensory cortices, thalamus, amygdala and the orbitofrontal cortex (Green et al., 2013). These results were followed by the related result that in autism there is decreased adaptation/habituation to sensory stimulation in the sensory cortex and the amygdala (Green et al., 2015). Both our finding that high-dose Vitamim-B6 reduces SOR and our proposal that it does this by influencing the E-I balance in favour of inhibition are consistent with these brain imaging results because although adaptation and habituation to sensory stimuli are both complex processes, neural inhibition and GABA are implicated in both (Larkin et al., 2010; Puts et al., 2014). Furthermore, a Vitamin-B-rich dietary intervention (marmite) has been shown to reduce neural responses measured by steady-state visual evoked potentials in primary sensory cortices (Smith et al., 2017), and so it is likely that our intervention had a similar effect, which future brain imaging studies could measure.

### Reduction in PD

Vitamin-B6 caused a selective reduction in scores on the PD subscale of the SP-3D. This was made up of seven questions that focused on whole-body strength, endurance, balance and posture, as well as coordination across multiple limbs, bimanual coordination and eye movement control. Considering previous findings emphasising the relationship between this type of motor function and neural inhibition/GABA, this effect could also be explained by Vitamin-B6 increasing GABAergic tone in the brain. Bimanual coordination and coordination across multiple limbs both depend critically on communication between the two brain hemispheres via the corpus callosum (Johansen-Berg et al., 2007). Suggesting a specific role of GABAergic inhibition in this, in Developmental Coordination Disorder, the inhibitory aspect of this communication is selectively reduced while intrahemispheric motor cortex inhibition is normal (Blais et al., 2018; He et al., 2018). Furthermore, GABA levels in the motor cortex correlate with bimanual coordination in an age-dependent way (Maes et al., 2021). One study has linked Vitamin-B6 to whole-body aspects of motor coordination, suggesting that this may be particularly sensitive to Vitamin-B6 status, it is generally considered that 8 weeks of dietary deprivation induces moderate deficiency in rats (Dakshinamurti et al., 1992), yet Schaeffer et al. (1990) found that deprived rats displayed gait disturbances after only 2–3 weeks.

### Other aspects of sensory reactivity

As well as the PD subscale, the SP-3D contains a related dyspraxia subscale. Scores on this subscale fell at post-test in both the Vitamin-B6 and placebo conditions, which in the experimental design we used does not provide evidence that Vitamin-B6 is beneficial. Equally, these results do not rule out such an effect, and a larger formal clinical trial is required to answer this question definitively. The PD and dyspraxia subscales are moderately correlated (Table 2), and their correlation is stronger than that between the other subscales of the SP-3D except SUR and SC. However, the dyspraxia subscale differs from the PD subscale in containing questions that tap mainly into higher-level cognitive and planning aspects of motor control, fine motor control, motor learning and speech production. It would be theoretically informative if a future study were to confirm a dissociation between the effects of Vitamin-B6 on the two different aspects of motor control assessed by the PD and dyspraxia subscales.

We found no selective effects of Vitamin-B6 on the other sensory subscales of the SP-3D: sensory under-responsivity, sensory craving and sensory discrimination. Scores on these subscales were generally reduced at post-test, but this occurred in either two, or all three of, the experimental groups. It is likely that regression to the mean, which can occur in any experimental design that selects participants on the basis that they score highly on a given trait or state, contributed to these effects (Barnett, 2005).

### Sensory reactivity in different groups of people

Most previous studies of SOR have focused on autistic participants, despite the literature cited in the introduction of this paper indicating that it also co-occurs with ADHD and other conditions, as well as occurring alone. Participants in the present study were not screened for these commonly co-occurring conditions, but it is reasonable to assume that our participant sample was mixed. Given the complexity of sensory processing, it is possible that the underlying causes of hyperreactivity are heterogeneous. Suggesting that this is the case, we found a different pattern of co-occurrence of the features of sensorimotor difference as measured by the SP-3D compared to the pattern found in studies of sensory differences in autistic participants. In autism, MacLennan et al. (2021) found that the co-occurrence of SOR with SC was high, while we found that this was low, and while we found that the co-occurrence of SUR and SC was very high, MacLennan et al. (2021) found that this was low in autism. This difference in findings is most easily accounted for by the different populations sampled in the two studies—autism versus all individuals experiencing hyperreactivity. The high-dose Vitamin-B6 intervention may prove more beneficial for some of the subgroups experiencing hyperreactivity than others, although because its effectiveness relies on a ubiquitous neural mechanism it is also possible that all individuals experiencing SOR and related conditions will benefit.

We found no evidence that SOR was more prevalent in either males or females in our sample, yet interestingly the reduction in SOR achieved by the high-dose B6 intervention was greater in males, although still reliable in females. This observation requires replication, and it would also be interesting to determine whether the B6 intervention is more effective in people whose SOR accompanies a diagnosis of autism, ADHD, other conditions, or occurs alone.

The majority of our sample had lower SOR scores in the neurotypical range and were not the main focus of the study. However, to check our assumption that the high-dose B6 intervention would only have measurable effects in the subgroup with high SOR scores, we performed an additional analysis in this group. This confirmed that there were no significant reductions in SOR scores in this group, and the presence of a slight trend towards a reduction was similar in the placebo, B6 and B12 groups, rather than selective to B6. Turning to the reduction of PD scores by B6, this also was non-significant in all three experimental conditions for the low PD group, although B6 specifically did trend towards significance in this case. Analysis of the remaining subscales of the SP-3D in participants with lower scores also revealed no effects. Taken together with our main analysis, this suggests that the effects of high-dose B6 at the behavioural and psychological level are more likely to be detectable in participants whose scores fall in the tails of the population distribution.

### Limitations

We have focused on the role of Vitamin-B6 as the crucial coenzyme for the conversion of glutamate to GABA by the enzyme GAD because this is the most plausible mechanistic explanation of our results. However, we did not measure GABA and glutamate concentrations in the brain directly, and because it is well established that Vitamin-B6 is a co-factor for over 100 metabolic reactions, some of which affect neural activity, we cannot rule out that effects on other metabolic pathways contributed to our results. For example, several tryptophan-related metabolic pathways are Vitamin-B6 dependent, including conversion of 5-HTP to serotonin; due to this, Vitamin-B6 depletion has been used as a model of serotonin deficiency in rats (Dakshinamurti et al., 1992). The synthesis of the neurotransmitters, such as dopamine, norepinephrine, histamine, and taurine, is B6-dependent (Kennedy, 2016; Rucker et al., 2007).

It would have been informative to compare the scores on the SP-3D subscales of the participants, we selected for analysis with published norms for groups that are known to experience SOR and other sensory-related problems, such as autism and ADHD. This was not possible because we were unable to find published norms for the self-completion version of the SP-3D questionnaire we used. The most similar version of the scale for which norms are available is completed by parents about their child, and it contains a far greater number of questions because each one asks about the presence of one highly specific example (Schoen et al., 2016). Whereas, in the version with fewer items, we used each question can be endorsed by the participant if one or more of a list of examples is experienced. Because of these differences, the psychometric properties of the two forms of the scale are not comparable. Having said that, participants in our high SOR group endorsed a minimum of 8/12 questions, which suggests that SOR was a notable feature of their lived experience and outside the neurotypical range.

The absolute levels of self-reported SUR, SC, dyspraxia and SD in the subgroups of the full sample that we selected for statistical analysis were not as high as the absolute levels of SOR and PD reported by the participants in the subgroups selected for those traits. Therefore, our data provided a stronger test of Vitamin-B6 efficacy for SOR and PD than the other subscales of the SP-3D.

We did not collect blood samples so could not quantify the Vitamin-B6 status of participants. Therefore, we were not able to verify that participants consistently took the tablets during the supplementation period or that circulating levels of Vitamin-B6/12 increased. If the data we analysed included some participants whose compliance was poor, then the effect sizes we report are likely to be underestimated, which would not undermine our main conclusions. Also because of this, we were unable to plot the correlations between baseline Vitamin-B6 status and baseline SOR, etc., or determine whether responding to the treatment was related to moderate B6 deficiency at baseline.

## Conclusion

We have shown that high-dose Vitamin-B6, an intervention that increases GABA and reduces glutamate concentration in animal models, reduces sensory hyperreactivity. To determine whether high-dose Vitamin-B6 is beneficial in all cases of SOR, or only when SOR occurs together with specific conditions, clinical trials should be conducted. Given that the probable mechanism of action-altering E-I neural balance – is likely to be beneficial in a range of conditions, trials should include a broad range of outcome measures in addition to SOR. Trials should be designed to determine whether some individuals respond strongly to high-dose B6 treatment and others do not, because there are reasons to expect this. Firstly, it is possible that individuals whose diets are moderately or severely B6-deficient will show larger treatment effects. Secondly, those individuals whose ability to metabolise inactive dietary forms of Vitamin-B6 to produce the active form is reduced are particularly likely to benefit. For example, there is evidence that this is the case in a subset of autistic individuals (Audhya, 2004; Adams et al., 2011; Obara et al., 2018), and there are also substantial individual differences in Vitamin-B6 metabolism in the general population that could result in some individuals being more responsive to high-dose B6 than others (Hadstein and Vrolijk, 2021; Vrolijk et al., 2020). Finally, it is important to note that we see the potential of this intervention as being complimentary to a social-model approach to neurodiversity (e.g., Woods, 2017), in which environments, language and attitudes are modified.
